# Delays in diagnosis and treatment of ATTR cardiac amyloidosis: A real‐world data analysis

**DOI:** 10.1002/ehf2.15311

**Published:** 2025-04-28

**Authors:** Julia Vogel, Sophia Jura, Stephan Settelmeier, Florian Buehning, Tobias Lerchner, Alexander Carpinteiro, Tienush Rassaf, Lars Michel

**Affiliations:** ^1^ Department of Cardiology and Vascular Medicine, West German Heart and Vascular Center University Hospital Essen Essen Germany; ^2^ West German Amyloidosis Center University Hospital Essen Essen Germany; ^3^ Department of Hematology and Stem Cell Transplantation, West German Cancer Center University Hospital Essen Essen Germany

**Keywords:** amyloidosis, ATTR, cardiac amyloidosis, diagnosis, tafamidis, transthyretin

## Abstract

**Aims and Background:**

Cardiac amyloidosis leads to functional cardiac impairment and heart failure. Transthyretin amyloid cardiomyopathy (ATTR‐CM) is the most common form. After initial suspicion, diagnosis involves imaging techniques, biopsy and genetic tests, prompting transthyretin stabilizer therapy to slow disease progression. The study aims to assess delays in diagnosis in ATTR‐CM patients.

**Methods:**

Patients with ATTR‐CM receiving transthyretin stabilizer therapy at the West German Amyloidosis Center (01/2018–12/2023) were included. Clinical, laboratory, and imaging data were analysed. Diagnostic timelines were compared across two periods (2018–2020 and 2021–2023).

**Results:**

After screening 254 patients, 154 were included in the analysis. ATTRwt was the most common form (96.8%). The median age was 80 (76–83) years, 87% were male and 46.6% were NYHA class ≥III. Time to diagnosis decreased from 398 to 277 days in the second period (*P* < 0.001). The median duration from diagnosis to stabilizer therapy was 84 (44–160) days, reducing from 111 (55–237) days in the first period to 57 (36–102) days in the second period (*P* < 0.001). Patients diagnosed in the first period had lower LVEF (*P* < 0.001) and more advanced NAC stages (*P* = 0.004). More women were diagnosed in the second period (*P* = 0.010).

**Conclusion:**

ATTR‐CM is associated with diagnostic delays from initial suspicion to therapy initiation. While diagnostic and treatment timelines have improved, enhanced awareness, supraregional networks, specialized centres and focused education are essential to improve diagnosis and outcomes. Increasing awareness has led to patients being diagnosed at earlier disease stages, underscoring the potential to positively impact patient prognosis.

## Introduction

Cardiac amyloidosis (CA) is a severe condition characterized by abnormal protein deposition in the heart, leading to functional impairment and heart failure.^1^ Symptoms suggestive for heart failure, an intraventricular septal thickness (IVSD) of ≥12 mm together and presence of ≥1 *red flag* sign/symptom should prompt further screening of CA.^2^ *Red flag* signs/symptoms include peripheral neuropathy, carpal tunnel syndrome, biceps tendon rupture and spinal stenosis among others.^1^ Transthyretin amyloid cardiomyopathy (ATTR‐CM) is the most common CA form and primarily affects older individuals, with men being more frequently affected than women.^3^ ATTR‐CM can present as wild‐type (ATTRwt‐CM) or variant ATTR‐CM (ATTRv‐CM).^4^ Transthyretin stabilizer therapy (e.g., tafamidis) is the only approved treatment so far to slow the progression of the disease,^5^ but several new substances are in late clinical development.^6^, ^7^ A delayed diagnosis of ATTR‐CM leads to worsening of the disease course with worse treatment outcomes for patients.

### Aims

This study aims to evaluate the diagnostic delays from initial suspicion to therapy initiation in ATTR‐CM and to examine how these delays have changed over time.

### Methods

All patients ≥18 years diagnosed with ATTR‐CM at the West German Amyloidosis Center, University Hospital Essen, Germany, from January 2018 to December 2023 were screened for inclusion. Patients who did not receive t or patients with insufficient data on first sign/symptom were excluded from the primary endpoint analysis. The individual characteristics of excluded patients, including the reasons for not initiating stabilizer therapy, were systematically assessed in an extended analysis. Diagnosis was established through scintigraphy and endomyocardial biopsy at diagnostic uncertainty or abnormal laboratory findings according to the proposed algorithm of the 2021 position paper of the European Society of Cardiology followed by genetic testing to differentiate between ATTRv and ATTRwt.^1^ A comprehensive set of parameters was collected including laboratory values, clinical symptoms and echocardiographic measurements. Data were systematically gathered from the clinic's internal data system. Laboratory values assessed included a wide range of biomarkers relevant to diagnosis and progression of CA. Clinical symptoms were extracted from documented patient interviews and clinical examinations. Echocardiographic parameters were obtained from primary imaging data. Measurements included left ventricular ejection fraction (LVEF), left ventricular wall thickness, diastolic function, and stroke volume (SV), among others. Abnormal findings in laboratory and echocardiographic parameters indicative of CA were defined according to clinical recommendations.^1^ To assess changes in diagnosis over time, a comparison was made between two time periods, 2018–2020 and 2021–2023. Follow‐up continued until the last documented medical contact, as recorded in available medical records up to January 2025. Kaplan–Meier analysis was performed to assess a combined endpoint of all‐cause mortality and unplanned heart failure hospitalization, stratifying patients into two groups based on the time from the first suggestive sign or symptom to the initiation of stabilizer therapy.

A two‐sided *P* value of <0.05 was defined for statistical significance. Kolmogorov–Smirnov test was used to assess normal distribution for continuous data. Simple linear regression was performed for time from suspicion to diagnosis. Trends in diagnosis time, defined as the period from the initial suspicion symptoms of a possible amyloidosis to the definitive diagnosis, was analysed over two time periods of 3 years each, using the Mann–Whitney *U* test for continuous variables and *χ* ^2^ tests for dichotomous variables. Kaplan–Meier analysis was assessed by the log‐rank test. Suspicion in this context refers to any documented mention in the patient record of heart failure symptoms, abnormal echocardiography findings (defined as a septal thickness >12 mm) or incidental findings in other imaging modalities such as scintigraphy or magnetic resonance imaging (MRI), conducted for non‐cardiac indications, that raised the possibility of amyloidosis and prompted further diagnostic evaluation. The exact date was taken as the point when these symptoms or findings were first documented in the patient's medical record, marking the onset of suspicion for ATTR‐CM. Normally distributed variables were shown as mean ± standard deviation. Non‐normally distributed variables were documented as median (quartile 1–3). GraphPad Prism 10 (GraphPad Software, Boston, USA) and Excel (Microsoft, Redmond, USA) were used for data analysis. This study was approved by the ethics committee of the University Duisburg‐Essen (23‐11500‐BO).

## Results

A total of 254 patients were screened. Sixty patients were excluded due to the absence of stabilizer therapy initiation, and an additional 40 were excluded due to insufficient data including cases where the first symptom could no longer be identified, resulting in a final cohort of 154 patients. Among the 60 patients who did not receive stabilizer therapy, the reasons for non‐treatment were as follows: 21 patients did not return to the centre after diagnosis, 12 patients had no cardiac symptoms or no clear indication for therapy, 10 patients received an alternative therapy for ATTRv‐CM due to a predominant neuropathy phenotype, 16 patients were considered too frail or in a palliative situation and 1 patient had undergone heart transplantation (making stabilizer therapy unnecessary). Additional analyses did not show a significant change of time from first sign or symptom to initiation of stabilizer therapy on key baseline criteria including LVEF, N terminal pro brain natriuretic peptide (NT‐proBNP), high‐sensitive cardiac troponin I (hs‐cTnI) and IVSD (all *P* ≥ 0.05; *Figure* S1).

Almost all patients had the wild‐type form of ATTR‐CM (96.8%) and only five patients (3.2%) in this study had ATTRv‐CM. Staging was performed according to National Amyloidosis Centre (NAC) ATTR stages^8^ (*Table* 1). Median age of the group was 80 (76–83) years, 87% were male patients. Of the patients, 53.4% were in NYHA class I + II and 46.6% in NYHA class III + IV. The median NT‐proBNP was 3205 (1946–5636) pg/mL and hs‐cTnI was 45 (27–69) ng/L. Detailed baseline characteristics are shown in *Table* 1. Patients with NYHA class III/IV were older (*P* = 0.026), had more atrial fibrillation (*P =* 0.001), higher NT‐proBNP at baseline (*P* = 0.002) and follow‐up (*P* = 0.024) and lower stroke volume at follow‐up (*P* = 0.019) compared with those with NYHA class I/II (*Table* S1).

**Table 1:** Baseline characteristics.

Variable	All (*n* = 154)	2018–2020 (*n* = 72)	2021–2023 (*n* = 82)	*P* value
Age (years)	80 (76–83)	80 (76–82)	80 (77–83)	0.237
Male, *n* (%)	134 (87)	68 (94.4)	66 (80.5)	0.010*
BMI (kg/m^2^)	26.5 ± 4	25.3 ± 3.6	27.6 ± 4.1	<0.001*
NYHA, *n* (%)				
NYHA 1 + 2	78 (53.4)	31 (47.0)	49 (61.3)	0.084
NYHA 3 + 4	68 (46.6)	35 (53.0)	31 (38.7)	
Comorbidities				
Coronary artery disease, *n* (%)	72 (46.8)	35 (48.6)	37 (45.1)	0.433
Atrial fibrillation, *n* (%)	105 (68.2)	50 (69.4)	55 (67.1)	0.315
Diabetes mellitus, *n* (%)	43 (27.9)	22 (30.6)	21 (25.7)	0.683
COPD and/or asthma, *n* (%)	22 (14.3)	14 (19.4)	8 (9.8)	0.087
Type of amyloidosis, *n* (%)				
ATTRwt	149 (96.8)	69 (95.8)	80 (97.6)	0.546
ATTRv	5 (3.2)	3 (4.2)	2 (2.4)	
Diagnosis, *n* (%)				
Endomyocardial biopsy	47 (30.5)	32 (45.7)	15 (17.9)	< 0.001*
Scintigraphy	107 (69.5)	38 (54.3)	69 (82.1)	
Staging, *n* (%)				
NAC < 2	64 (41.6)	21 (29.2)	43 (52.4)	0.004*
NAC ≥ 2	90 (58.4)	51 (70.8)	39 (47.6)	
Haemodynamic				
Blood pressure mean (mmHg)	103 ± 16	100 ± 15	105 ± 16	0.073
Heart rate (bpm)	75 (65–87)	72 (64–81)	76 (65–88)	0.262
Laboratory values				
hs‐cTnI (ng/L)	45 (27–69)	51 (35–77)	41 (25–68)	0.088
NT‐proBNP (pg/mL)	3205 (1946–5636)	4200 (2739–8010)	2882 (1501–4768)	<0.001*
Creatinine (mg/dL)	1.2 (1–1.4)	1.2 (1–1.5)	1.1 (1–1.4)	0.757
eGFR (mL/min/1.73 m^2^)	58.4 ± 17.8	59 ± 19.4	57.8 ± 16.3	0.661
CRP (mg/dL)	0.4 (0.4–0.5)	0.5 (0.4–0.7)	0.4 (0.4–0.5)	0.025*
Hb (g/dL)	13.3 ± 1.6	13.1 ± 1.6	13.6 ± 1.6	0.073
INR	1.1 (1.0–1.2)	1.1 (1.0–1.2)	1.1 (1.0–1.2)	0.291
Echocardiography				
LVEF (%)	52 (43–55)	50 (40–55)	55 (46–55)	<0.001*
Stroke volume (mL)	52 (45–65)	52 (42–60)	54 (45–67)	0.411
LVM (g)	316 (270–368)	303 (268–359)	340 (276–399)	0.198
IVSD (mm)	18.1 ± 4.4	17.8 ± 4.6	18.2 ± 4.2	0.587
E/e′ ratio	13 (10–17)	13 (11–17)	14 (10–16)	0.898
GLS (%)	−10.6 ± 4.4	−11.3 ± 5.1	−10.1 ± 3.9	0.513

Abbreviations: ATTRv, variant ATTR; ATTRwt, wild‐type ATTR; BMI, body mass index; COPD, chronic obstructive pulmonary disease; CRP, C‐reactive peptide; eGFR, estimated glomerular filtration rate; Hb, haemoglobin; hs‐cTnI, high‐sensitive cardiac troponin I; INR, international normalized ratio; IVSD, interventricular septum thickness; LVEF, left ventricular ejection fraction; LVMM, left ventricular mass; NAC, National Amyloidosis Centre; NT‐proBNP, N terminal pro brain natriuretic peptide; NYHA, New York Heart Association.

* highlights *P* < 0.05.

**Figure 1:**
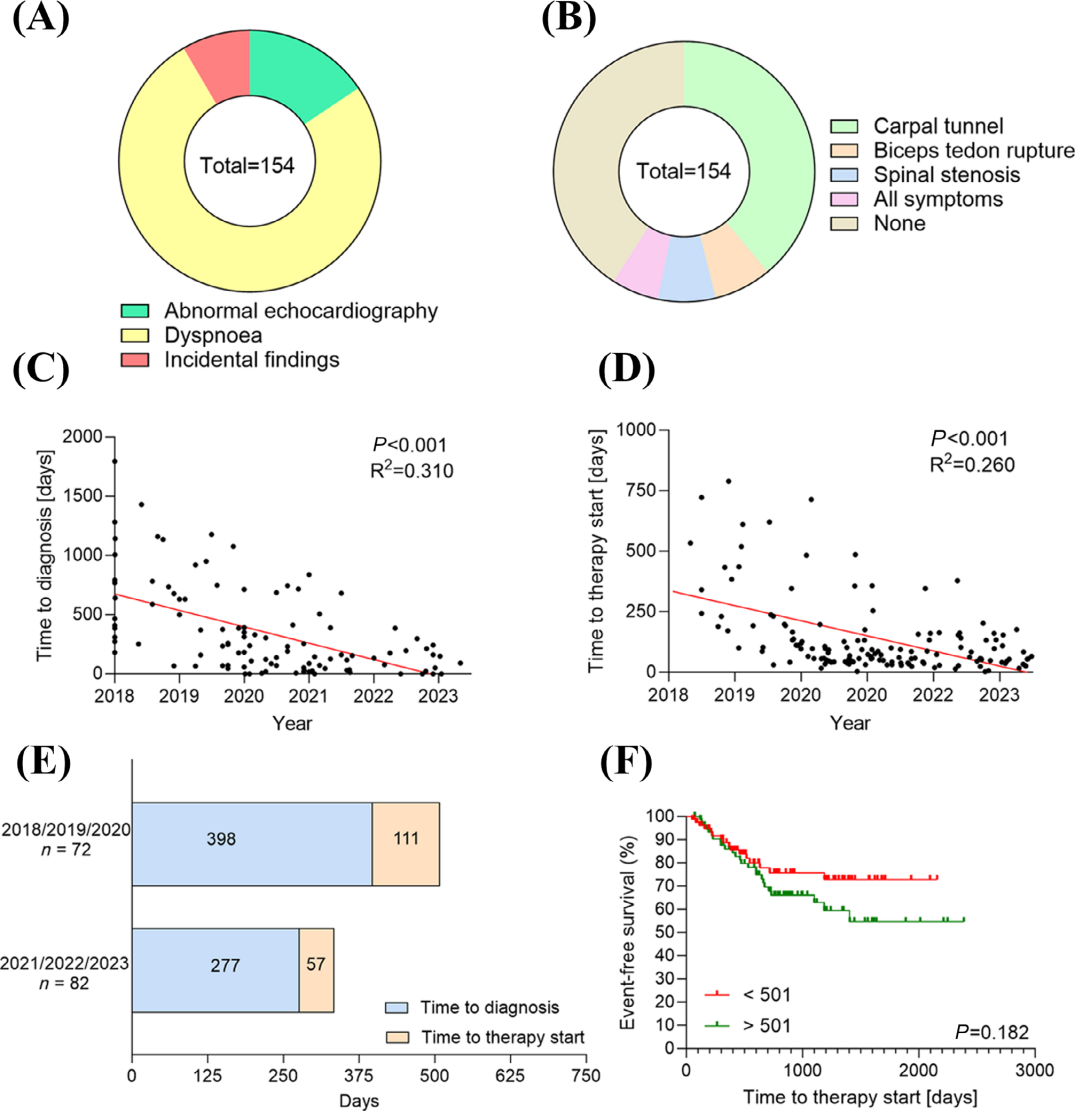
Initial cardiac (A) and non‐cardiac (B) symptoms/diagnostic with indications of transthyretin amyloid cardiomyopathy (ATTR‐CM). (C) Simple linear regression with correlation between days to diagnosis and date of suspicion criteria. (D) Simple linear regression with correlation between days to therapy start and date of diagnosis. (E) Time from suspicion to diagnosis and to prescription of tafamidis (F) Kaplan–Meier analysis to evaluate the combined endpoint of all‐cause mortality and unplanned heart failure hospitalization, stratified for patient groups based on longer versus shorter time from the first suggestive sign or symptom to the initiation of stabilizer therapy. Statistical comparisons were conducted using the log‐rank test.

The overall median time from suspicion to diagnosis of ATTR‐CM was 361 (99–957) days. The most frequent initial cardiac symptom that led to diagnosis or further diagnostic evaluation was unexplained dyspnoea, occurring in 117 patients (76%), followed by abnormal echocardiography in 24 patients (15.6%) and incidental findings in 13 patients (8.4%) (*Figure* 1A). Among the incidental findings, 1 case was identified through MRI and 12 through scintigraphy. Most of the scintigraphies (*n* = 7) were performed for orthopaedic indications, followed by prostate cancer staging (*n* = 3), while the reason for imaging in the remaining cases could not be clearly determined. Apparent *Red flag* signs/symptoms were reported in 59.1% of all patients with carpal tunnel (39%) as the most common finding (*Figure* 1B). Diagnosis time decreased from 398 (143–828) days in the first period (2018–2020) to 277 (69–1091) days in the second period. Linear regression analysis showed a decrease in days to diagnosis (*P <* 0.001) and between days from diagnosis to therapy prescription over time (*P* < 0.001) (*Figure* 1C–E).

The median duration from diagnosis to the prescription of transthyretin stabilizer therapy was 84 (44–160) days. When comparing the first period to the second period, a reduction in this duration was observed. Specifically, the median duration in the first period was 111 (55–237) days, whereas in the second period, it was reduced to 57 (36–102) days (*P* < 0.001) (*Figure* 1C). In the extended analysis, we assessed the time from diagnosis to initiation of stabilizer therapy in all patients that were initially excluded due to a lack of data on the initial symptom and/or time of symptom. These patients (*n* = 40) had a median age of 78 (73–82) years, and 85% were male. The median duration from diagnosis to therapy initiation was 186 (48–386) days in the first period, and 83 (61–169) days in the second period (*P* = 0.251).

In addition to the observed decrease in time to diagnosis and therapy initiation, differences in laboratory values and echocardiographic parameters were found between the two time periods. Patients in the earlier period (2018–2020) presented with higher NT‐proBNP levels and lower LVEF (both *P* < 0.001), suggesting more advanced cardiac involvement at diagnosis compared with those diagnosed in the second period (2021–2023). Further, when assessing disease severity using NAC staging, patients from the first period were more frequently in advanced stages at diagnosis, according to NAC staging (*P* = 0.004). In the second period, there was an increase in the proportion of female patients (*n* = 16, 19.5%) diagnosed with ATTR‐CM, as compared with the first period (*n* = 4, 5.6%) (*P* = 0.010). Additional analyses did not show a significant change of time from first sign or symptom to initiation of stabilizer therapy on key baseline criteria including LVEF, NT‐proBNP, hs‐cTnI and IVSD (all *P* ≥ 0.05; *Figure* S1). Over a median follow‐up of 668 (336–1216) days, Kaplan–Meier analysis targeting a combined endpoint of overall death and unplanned hospitalization for HF did not show a significant improvement in patients with a shorter time to therapy compared with those with a longer time to therapy (*P* = 0.182; *Figure* 1F). The overall mortality of patients was 2.6% (4 patients), thus indicating a comparably healthy cohort. Of these, two patients died of non‐cardiac cause (COVID‐19, solid cancer) and one patient from an unknown cause.

In line with potential sex‐specific diagnostic challenges, female patients exhibited higher LVEF at baseline compared with male patients (*P* = 0.035) and had a lower prevalence of coronary artery disease (*P* = 0.037). There was no difference in median IVSD. During follow‐up, no differences were observed between sexes regarding echocardiographic parameters, laboratory values, or NYHA class (Table S2).

**Figure 2:**
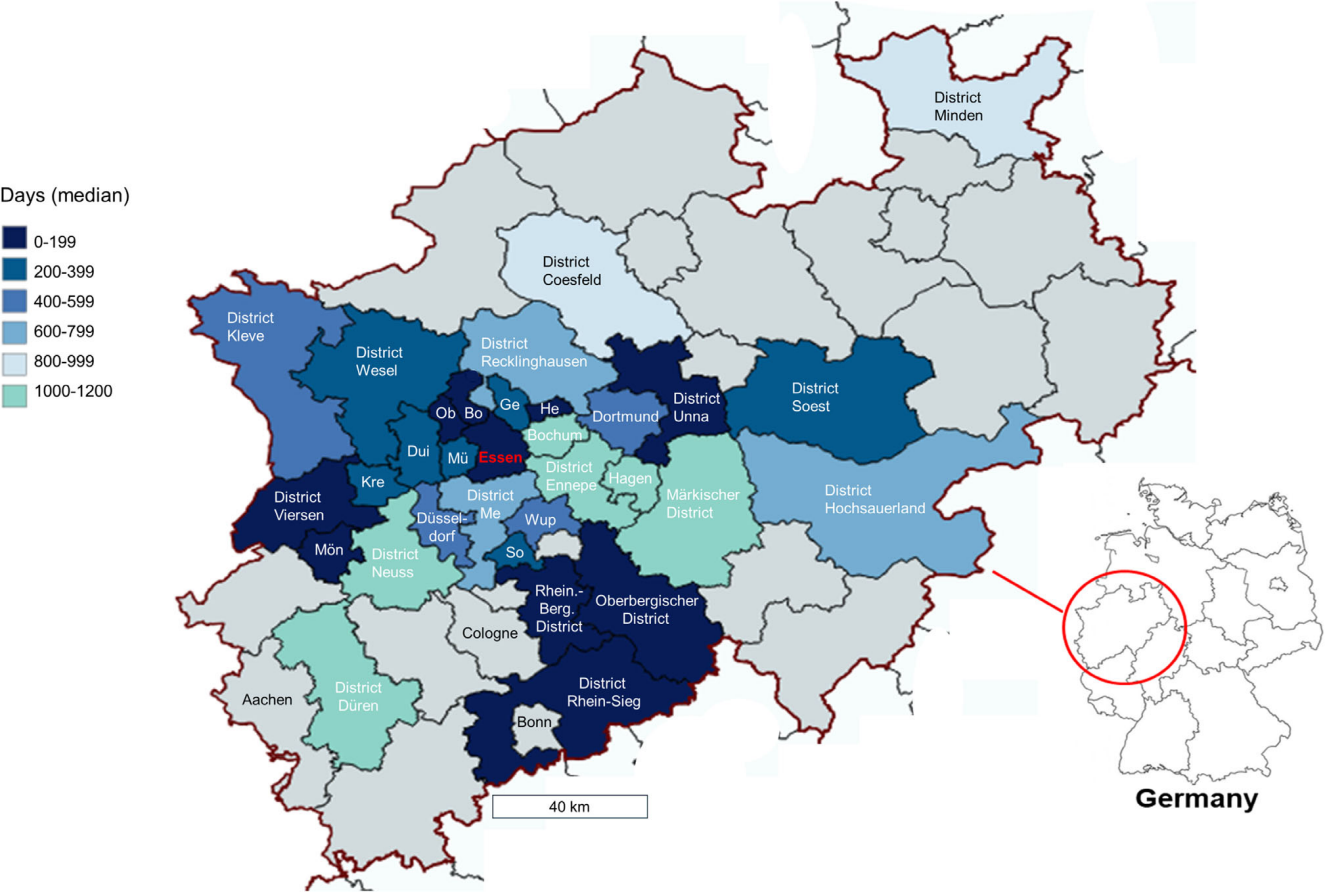
Average duration from suspicion to diagnosis in days stratified by cities/districts of residence in the state of North Rhine‐Westphalia, Germany. All patients represented in this figure were diagnosed and treated at the West German Amyloidosis Center, University Hospital Essen, Germany. Bo, Bottrop; Dui, Duisburg; Ge, Gelsenkirchen; He, Herne; Kre, Krefeld; Me, Mettmann; Mü, Mülheim; Ob, Oberhausen; So, Solingen; Wup, Wuppertal.

Furthermore, we observed that patients residing further away from our centre may experience a further delay in receiving treatment for ATTR‐CM. We investigated the distance from which patients with a short duration from suspicion to diagnosis lived and found a significantly shorter distance (direct line from city or district centre to Essen) compared with patients whose diagnosis took longer. Specifically, patients with a median time from suspicion to diagnosis of 0–199 days lived significantly closer to Essen than those with a median time of 800–999 days (*P* = 0.034). Similarly, patients with a median time to diagnosis of 200–399 days also lived closer than those with a median time of 800–999 days (*P* = 0.023) (*Figure* 2).

## Discussion

This study evaluated the time from symptom onset to ATTR‐CM diagnosis and the subsequent time to treatment initiation, revealing significant diagnostic delays. These delays are likely multifactorial, involving limited disease awareness—especially in the initial year following tafamidis approval—and symptom overlap with other diseases. Notably, tafamidis approval itself has spurred diagnostic efforts in the field. Our findings align with previous real‐world data, which emphasized the need for earlier diagnosis and highlighted persistent delays despite evident disease symptoms, underscoring the importance of improved screening strategies.^9^, ^10^, ^11^

In addition to these time improvements, notable shifts between the two periods were observed. Patients diagnosed in the first period had higher NT‐proBNP levels and lower LVEF, indicating more advanced cardiac dysfunction at diagnosis, which is also reflected in higher NAC stages. An interesting shift in gender distribution was noted. In the second period, there was an increase in the proportion of female patients as compared with the first period. These findings unmask an inherent risk of underdiagnosing female patients. As CA screening parameters (e.g., IVSD ≥ 12 mm) were predominantly based on data from male patients, this underscores an urgent need for a reevaluation of screening criteria to ensure they are inclusive of gender‐based variations.

Our data demonstrate not only a significant reduction in the time to diagnosis of ATTR‐CM but also that patients are being identified at substantially earlier disease stages. This finding highlights the success of efforts in recent years to increase awareness of ATTR‐CM. These efforts include targeted education for physicians, broader availability of modern diagnostic tools (e.g., scintigraphy and genetic testing), and a growing clinical awareness of ATTR‐CM. Education on specific signs and symptoms has increased significantly and remains essential, supported by regional training, specialized networks and interdisciplinary amyloidosis boards to streamline patient referral and diagnosis. At the time of the study, there were regular educational events on ATTR‐CM in the catchment area, including symposia, conferences and congress presentations. While an increase in referrals was observed over time, it remains unclear whether this was primarily due to these educational initiatives or a general rise in awareness.

## Limitations

The study exhibits relevant limitations. There is a risk of reporting bias from insufficient or incorrect reporting of initial signs/symptoms. The analysis is ultimately based on data on medical care in the Ruhr area, a very densely populated region with extensive medical coverage. The Ruhr area's healthcare infrastructure may particularly benefit elderly or immobile patients due to the proximity to medical facilities, which may not be the case in less urbanized areas. Together with the monocentric nature of the study, this represents a limitation, as findings may not be fully generalizable to other regions or healthcare systems. It is also important to consider that other amyloidosis centres are in different cities, which could impact our findings, as patients residing closer to these centres might receive an earlier diagnosis or treatment.

Furthermore, long‐term outcomes such as morbidity were not analysed in detail and should be addressed in future studies. The study also did not account for potential differences in socioeconomic status, nor did it explore differences in diagnostic delays based on ethnicity. The study's focus on cardiology reflects the typical pathway for ATTRwt‐CM, predominantly affecting older males with cardiac symptoms, explaining the male predominance and low ATTRv‐CM representation. Cases with neuropathic presentations, often treated with vutrisiran, follow different pathways and were less captured, limiting generalisability to younger or neuropathic patients.

## Conclusions

This study demonstrates significant progress in reducing diagnostic delays and improving treatment initiation timelines for ATTR‐CM. Patients are increasingly being diagnosed at earlier disease stages, reflecting the success of ongoing awareness campaigns, physician education and the availability of advanced diagnostic tools. These findings highlight the critical role of interdisciplinary networks, amyloidosis boards and specialized clinics in facilitating timely diagnosis and treatment access.

Despite these improvements, challenges remain. The observed underdiagnosis of female patients points to an urgent need to refine screening criteria to account for gender‐specific differences. Additionally, future studies should investigate the impact of diagnostic and treatment delays on patient outcomes, including mortality and morbidity. Understanding which specific interventions have been most effective will be crucial for replicating these successes on a broader scale and ensuring equitable access to care across diverse patient populations.

## Conflict of interest statement

Julia Vogel received personal fees outside the submitted work from Eli Lilly, Bayer and Pfizer and research funding from the UMEA Clinician Scientist Scholarship from the Faculty of Medicine, University of Duisburg‐Essen. Sophia Jura has no conflict of interest. Stephan Settelmeier received personal fees outside the submitted work from AstraZeneca and research funding from the UMEA Clinician Scientist Scholarship from the Faculty of Medicine, University of Duisburg‐Essen. Florian Buehning received research funding from the UMEA Clinician Scientist Scholarship from the Faculty of Medicine, University of Duisburg‐Essen. Tobias Lerchner has no conflict of interest. Alexander Carpinteiro received personal fees outside the submitted work from Alexion, Alnylam, Amgen, Janssen, Pfizer and Sanofi and research funding from the German Research Foundation (DFG; CA 2420/2‐1). Tienush Rassaf received personal fees outside the submitted work from Novartis, Bristol Myers Squibb, Bayer, Daiichi Sankyo, AstraZeneca and Pfizer and research funding from the German Research Foundation (DFG; RA 969/12‐1; RTG 2989). Lars Michel received personal fees outside the submitted work from Bayer, Alnylam, AstraZeneca, IFFM e. V. and from Bund der Niedergelassenen Kardiologen (BNK) and research funding from te IFORES Clinician Scientist Scholarship from the Faculty of Medicine, University of Duisburg‐Essen.

## Supporting information


**Table S1.** Baseline characteristics of NYHA classes.
**Table S2.** Baseline characteristics of sex differences.
**Figure S1.** Simple linear regression analysis with correlation of time from first sign or symptom to initiation of stabilizer therapy and (A) left ventricular ejection fraction (LVEF), (B) interventricular septal diameter (IVSD), (C) N‐terminal pro brain natriuretic peptide (NT‐proBNP) and (D) high‐sensitive cardiac troponin I (hs‐cTnI).
