# Supplementary material for: Delays in diagnosis and treatment of ATTR cardiac amyloidosis: A real‐world data analysis
Source: ESC Heart Fail. 2025 Apr 28;12(4):2969–75. doi: 10.1002/ehf2.15311 (PMC12287846; doi:10.1002/ehf2.15311)
Supplement: Supplementary file 1 — Table S1. Baseline characteristics of NYHA classes. Table S2. Baseline characteristics of sex differences. Figure S1. Simple linear regression analysis with correlation of time from first sign or symptom to initiation of stabilizer therapy and (A) left ventricular ejection fraction (LVEF), (B) interventricular septal diameter (IVSD), (C) N‐terminal pro brain natriuretic peptide (NT‐proBNP) and (D) high‐sensitive cardiac troponin I (hs‐cTnI). [file EHF2-12-2969-s001.pdf]

**Delays in diagnosis and treatment in ATTR cardiac amyloidosis: a real-world data analysis**

**SUPPLEMENTARY MATERIAL**

Julia Vogel, MD[a,b], Sophia Jura[a,b], Stephan Settelmeier, MD[a,b], Florian Buehning, MD[a], Tobias Lerchner, MD[a], Alexander Carpinteiro, MD[b,c], Tienush Rassaf MD[a,b], Lars Michel MD[a,b]*

[a] Department of Cardiology and Vascular Medicine, West German Heart and Vascular Center, University Hospital Essen, Hufelandstr. 55, 45147 Essen, Germany

[b] West German Amyloidosis Center, University Hospital Essen, Hufelandstrasse 55, 45147 Essen, Germany

[c] Department of Hematology and Stem Cell Transplantation, West German Cancer Center, University Hospital Essen, Hufelandstr. 55, 45147 Essen, Germany

*Corresponding author: Dr. Lars Michel, Department of Cardiology and Vascular Medicine, West German Heart and Vascular Center, University Hospital Essen, Hufelandstr. 55, 45147 Essen, Germany.

Mail: lars.michel@uk-essen.de, Fax: +492017235401 Tel: +4920172384841

**Supplementary Table S1:** Baseline characteristics of NYHA classes

| **Variable** | **NYHA I/II** (n=80) Baseline | **NYHA III/IV** (n=66) Baseline | **p-value** | **NYHA I/II** (n=60) 12 Months | **NYHA III/IV** (n=47) 12 Months | **p-value** |
|---|---|---|---|---|---|---|
| Male, n (%) | 69 (86.3) | 58 (87.9) | 0.771 | | | |
| Age (years) | 79 (76-82) | 81 (78-83) | 0.026* | | | |
| BMI (kg/m$^2$) | 25.8 (23.5-28.3) | 26.2 (23.5-29.1) | 0.475 | | | |
| Coronary artery disease, n (%) | 33 (41.3) | 34 (51.5) | 0.215 | | | |
| Atrial fibrillation, n (%) | 45 (56.3) | 54 (81.8) | <0.001*** | | | |
| | | | | | | |
| LVEF (%) | 54 (45-55) | 50 (41-55) | 0.302 | 50 (46-55) | 46 (41-55) | 0.258 |
| SV (ml) | 51 (43-67) | 52 (43-60) | >0.999 | 54 (40-67) | 41 (37-55) | 0.019* |
| Creatinine (mg/dl) | 1.1 (0.9-1.3) | 1.3 (1.0-1.5) | 0.117 | 1.2 (1.0-1.5) | 1.3 (1.1-1.7) | >0.999 |
| hs-cTnI (ng/l) | 41 (25-57) | 52 (34-92) | 0.071 | 33 (27-76) | 44 (22-227) | >0.999 |
| NT-proBNP (pg/ml) | 2882 (1300-4667) | 4359 (2854-7973) | 0.002** | 2750 (1394-4744) | 4176 (2565-7793) | 0.024* |

BMI, body mass index; hs-cTnI, high-sensitive cardiac troponin I; LVEF, left ventricular ejection fraction; NT-proBNP, n-terminal brain natriuretic peptide; NYHA, New York Heart Association; SV, stroke volume.

**Supplementary Table S2:** Baseline characteristics of sex differences

| **Variable** | **Male** (n=134) Baseline | **Female** (n=20) Baseline | **p-value** | **Male** (n= 101) 12 Months | **Female** (n=13) 12 Months | **p-value** |
|---|---|---|---|---|---|---|
| Age (years) | 80 (77-83) | 81 (76-84) | 0.592 | | | |
| BMI (kg/m$^2$) | 26.1 (23.5-28.4) | 25.4 (23.3-31.3) | 0.628 | | | |
| Coronary artery disease, n (%) | 67 (50.0) | 5 (25.0) | 0.037* | | | |
| Atrial fibrillation, n (%) | 92 (68.7) | 12 (63.2) | 0.631 | | | |
| | | | | | | |
| NYHA, n (%) | | | 0.771 | | | 0.376 |
| *1+2* | 69 (54.3) | 11 (57.9) | | 65 (66.3) | 7 (53.9) | |
| *3+4* | 58 (45.7) | 8 (42.1) | | 33 (33.7) | 6 (46.1) | |
| | | | | | | |
| LVEF (%) | 50 (42-55) | 55 (51-59) | 0.035* | 50 (45-55) | 55 (50-55) | 0.346 |
| SV (ml) | 54 (45-66) | 49 (42-59) | >0.999 | 48 (39-63) | 51 (36-75) | >0.999 |
| IVSd (mm) | 19 (15-21) | 16 (14-22) | >0.999 | 19 (17-21) | 18 (15-22) | >0.999 |
| Creatinine (mg/dl) | 1.2 (1.0-1.5) | 1.0 (0.8-1.2) | 0.050 | 1.3 (1.0-1.5) | 1.1 (0.9-1.5) | 0.955 |
| eGFR (ml/min/1,73m3) | 58.7 ± 18.1 | 55.9 ± 15.8 | 0.910 | 54.0 ± 17.7 | 49.3 ± 18.8 | 0.804 |
| hs-cTnI (ng/l) | 48 (27-70) | 30 (7-61) | 0.211 | 36 (21-65) | 25 (25-25) | >0.999 |
| NT-proBNP (pg/ml) | 3318 (2013-5987) | 2758 (899-4827) | 0.446 | 3446 (1962-6216) | 2223 (412-4371) | 0.272 |

BMI, body mass index; eGFR, estimated glomerular filtration rate; hs-cTnI, high-sensitive cardiac troponin I; IVSd, interventricular septum thickness; LVEF, left ventricular ejection fraction; NT-proBNP, n-terminal brain natriuretic peptide; NYHA, New York Heart Association; SV, stroke volume.

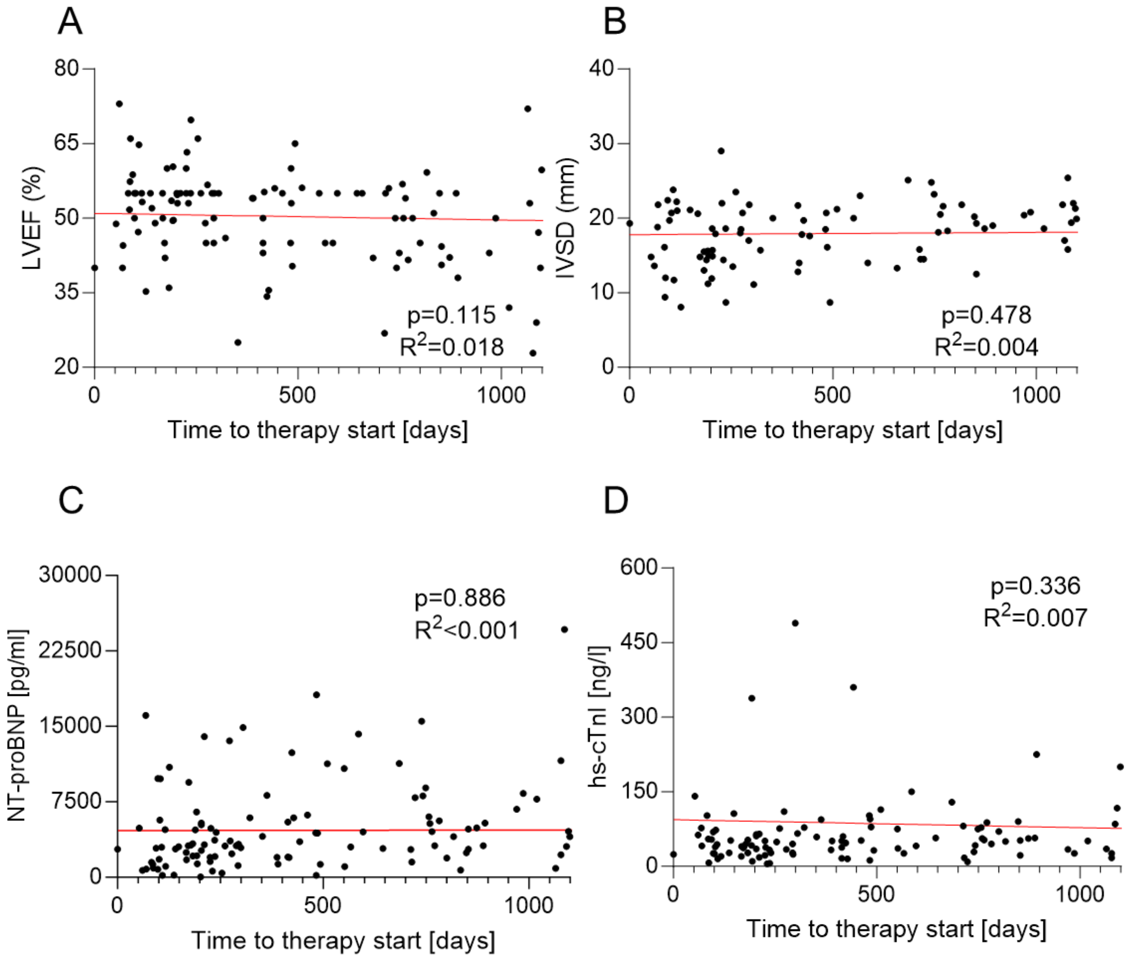


**Supplementary Figure S1.** Simple linear regression analysis with correlation of time from first sign or symptom to initiation of stabilizer therapy and (A) left ventricular ejection fraction (LVEF), (B) interventricular septal diameter (IVSD), (C) N-terminal pro brain natriuretic peptide (NT-proBNP) and (D) high-sensitive cardiac troponin I (hs-cTnI).
